# Observing Others’ Gaze Direction Affects Infants’ Preference for Looking at Gazing- or Gazed-at Faces

**DOI:** 10.3389/fpsyg.2018.01503

**Published:** 2018-08-13

**Authors:** Mitsuhiko Ishikawa, Shoji Itakura

**Affiliations:** Department of Psychology, Graduate School of Letters, Kyoto University, Kyoto, Japan

**Keywords:** eye gaze, face preference, joint attention, preferential looking, eye tracking

## Abstract

Eye gaze is an important signal in social interactions, and it plays an important role to understand what others looking in joint attention (JA) situations. JA has been examined in situations involving two people gazing at objects; however, ecologically, infants observe not only faces that gaze at objects but also those that gaze at other people. Here, we examined how eye gaze directed toward another face affect face preferences in infants. A total of 19 children were observed during a JA situation and a no-JA situation. In the JA situation, an adult face in the central position of the screen shifted her gaze to look at another adult face at a lateral position on the screen. However, during the no JA situation, the central face shifted her eye gaze away from the adult face presented on the screen. At test, for the centrally presented faces, infant looking times were longer at faces in the no JA condition. At test, for the laterally presented faces, looking times were longer at the faces in the JA condition. Thus, the adult’s eye gaze biased the duration of the gaze of the infants at either the central faces or the lateral-cued faces in the preferential looking tests. These results suggest that 10-month-old infants may interpret adult gazing behavior and that this can affect the gazing behavior of infants.

## Introduction

Many studies have demonstrated that the human eye gaze is one of the most important signals of non-verbal communication (Senju and Johnson, 2009). From as young as six months of age, infants look in the same direction as others are looking (D’Entremont et al., 1997). Gaze following ability supports infants’ engagement in joint visual attention, and it supports for the development of pointing, language acquisition, and the theory of mind (Bruner, 1983; Butterworth and Grover, 1990; Baron-Cohen, 1997). Eye gaze plays a critical role in directing and coordinating attention during triadic interactions between self, other, and the environment, especially during human social interaction (Grossmann et al., 2013). Such triadic interactions have been termed joint attention (JA) situations (Carpenter et al., 1998).

Various definitions have been used to refer to infant JA (Carpenter et al., 1998). During this study, we adopted the following definition of JA: looking in the same direction as someone else (Scaife and Bruner, 1975; Butterworth and Cochran, 1980). Two aspects of JA have been studied in infants; responses to the bidding of others and spontaneous initiations (Mundy and Newell, 2007). Responding to joint attention (RJA) refers to the ability of infants to follow the direction of another’s gaze to share a common point of reference; whereas initiating joint attention (IJA) refers to infants’ spontaneous use of their gaze to direct or share others’ attention to objects, events, and themselves. For example, during one study of RJA situations, the directions of others’ gazes were used for cuing (Reid and Striano, 2005). There is a difference in the development of RJA and IJA. As early as six months of age, infants display RJA behavior (Morales et al., 1998, 2000; Brooks and Meltzoff, 2005). However, IJA develops at nine to 10 months of age only (Venezia et al., 2004), and the frequency of IJA behavior does not increase between nine and 18 months (Mundy et al., 2007). Although many developmental studies have examined JA by observing infant behavior such as pointing (see Tomasello et al., 2007 for a review), screen-based tasks also have been used when investigating cognitive aspects of JA.

Screen-based studies have shown that infants have sensitivity for gaze shifts, which is an important component of RJA. Previous studies have used eye gaze cuing paradigm of Hood et al. (1998), which adopted from Posner’s cueing paradigm (Posner, 1980). Farroni et al. (2000) showed that infants shift their attention to peripheral targets cued by the direction of eye gaze of a central face. This eye gaze cueing paradigm has been used to examine its effects on preferences. First, studies of gaze cuing in RJA situations have revealed that adults prefer faces that engage in congruent gaze behavior (Bayliss and Tipper, 2006; Bayliss et al., 2009). Screen-based IJA tasks have also shown that IJA affects the preference for faces presented on the screen. Adults liked avatars more when the avatars followed their gaze contingently, and avatars’ gaze following increased adults’ positive evaluations of them (Grynszpan et al., 2017).

Both RJA and IJA situations affect the preference for gaze targets, such that the more people pay visual attention to an object the more other adults like the object (Bayliss et al., 2006). These JA effects have also been observed in infants. Okumura et al. (2013) set up infant RJA situation and examined preferences in 12-month-old infants using an object choice test during which they were shown two real objects, one that was looked at by another person during the initial phase and the other that was not. Infants reached for the gaze-cued object more often than the non-cued object. This result has also been demonstrated during a screen-based infant mock IJA situation (Ishikawa et al., unpublished).

Thus, these studies of JA situations have demonstrated two types of preference: for the gaze shifter (people who shift their gaze direction on the screen) and for the gaze target (objects which are looked at by gaze-shifter). Although JA has been examined primarily in triadic interactions of self-other-object, gaze targets in ecological situations may be faces as well as inanimate objects. One explanation of the gaze-shifter preference is that looking at the target increases trustworthiness. In one study, adults evaluated faces that looked at a target as more trustworthy than the faces that did not look at the target (Bayliss and Tipper, 2006). In addition, an infant study demonstrated that gaze following behavior might be highly related to the perceived reliability of lookers (Chow et al., 2008). 10-month-old infants already have the capacity to evaluate others’ traits from observing their behavior in social interactions (Hamlin et al., 2007).

Ecologically, infants observe not only faces that gaze at objects but also those that gaze at other people. Beier and Spelke (2012) investigated the understanding of infants of the social gaze in third-party interactions, and they showed that 10-month-old infants expect a person to look at their social partner during interaction. For example, when infants observe social gaze interactions between others (e.g., when infants meet strangers with their caregivers), it could be a JA situation (e.g., both the infant and a caregiver look at the stranger). Moreover, JA may affect the preference for gaze shifters and gaze targets (Okumura et al., 2013; Grynszpan et al., 2017). Therefore, the observation of social gaze interactions between others indicates that JA situations enhance a social preference for the person that is being looked at. We used a mock IJA paradigm during this study, to maximize eye gaze effects on social preferences, because IJA may have stronger effects than RJA may have (Grynszpan et al., 2017).

During this study, 10-month-old infants were presented with pictures of a central face turning her gaze either toward (joint attention situation; JA) or away (no joint attention situation; no-JA) from a lateral target face, which was presented to the left or right of the central face. In all the trials, the central face shifted her gaze when infants looked at a lateral target face and looked back to the central face. We checked that infants looked first at the central face, then, shifted to the lateral face and shifted back to the central face; therefore, we considered this as a mock IJA situation. Following this phase, the looking test presented two central faces and two target faces in a JA situation and no-JA situation. In a previous adult study that used a screen-based IJA situation, the participants preferred faces that shifted their gaze toward targets to faces that shifted their gaze away from targets (Grynszpan et al., 2017); therefore, we predicted that infants would display a preference for the JA central face, demonstrated by a longer gaze, over the no-JA central face. In addition, gaze cued targets were preferred during the adult study (Bayliss et al., 2006); therefore, we expected that infants would also prefer the JA lateral face over the no-JA lateral face.

## Materials and Methods

The experimental protocol was approved by the Research Ethics Review Board of the Department of Psychology, Kyoto University, Kyoto, Japan. The parents or caregivers of all the participants provided written informed consent before their infants participated in this study.

### Participants

The participants were nineteen 10-month-old infants (10 males, nine females; mean age 306.15 days; range 286–317 days). Infants are able to follow others’ gazes at this age (Gredebäck et al., 2008) and understand the relationship between the looker and the target (Woodward, 2003; Senju et al., 2008). Ten additional infants were excluded from the analyses due to inattentiveness (five infants who had less than six trials of gazing at the screen after the gaze shift during familiarization, and five infants who did not look at faces for at least two seconds during the looking test).

### Apparatus and Stimuli

**FIGURE 1:**
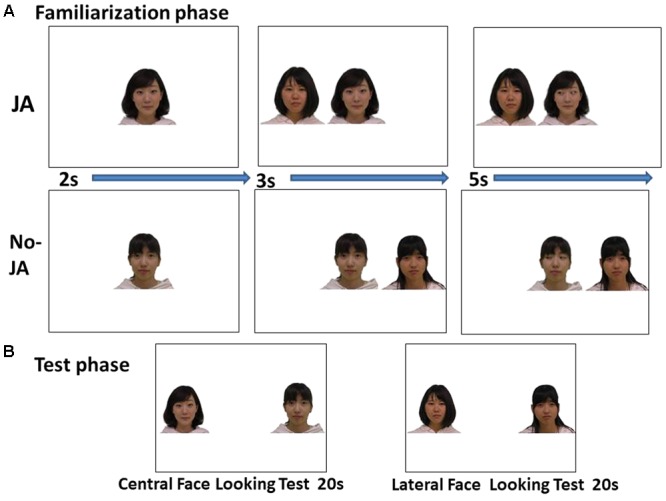
Stimuli used during the familiarization and test phases. **(A)** Illustration of the familiarization phase during the trial experiment. **(B)** Illustration of the test phases.

A Tobii T60 Eye Tracker integrated with a seventeen-inch TFT monitor was used to present stimuli and to record eye movements at a 60 Hz sampling rate (Tobii Studio 2.2.8, Tobii Technology, Stockholm, Sweden). The participants were seated in a caregiver’s lap approximately sixty centimeters from the monitor. Prior to the recording, a five-point calibration was conducted. **Figure 1** shows the stimuli used during the familiarization and test phases.

During the familiarization phase, all the faces were 5° wide and 7° high. Three kinds of faces were presented: a direct gaze face as a pre-cuing stimulus, and right- and left-gazing faces. In addition, a lateral face appeared approximately 10° to the left and right of the center. Four female identities were prepared for the study in total.

During the looking test phase, two central faces and two lateral faces were presented in successive pairs and in a counterbalanced order across participants.

### Procedure

We used a mock IJA situation and checked that infants looked first at the central face, then, shifted to the lateral face and, then, shifted back to the central face.

During the familiarization phase, infants viewed six trials in each situation (JA, No-JA). The central face with a direct gaze appeared initially during the familiarization phase (2 s). Second, a face appeared to either the left or the right of the central face (3 s). Third, the central face shifted its gaze to the left or the right. In other words, the central face either turned her gaze toward (JA) or away from (No-JA) the lateral face. The central female shifted her gaze direction, so was named the gaze-shifter. The target’s location changed in an ABBABA order. The presentation of target faces and gaze-shifter faces during each situation was counterbalanced across participants. All the facial stimuli maintained a neutral facial expression. The familiarization phase comprised twelve trials. Five participants who did not engage during at least six trials of the familiarization phase were excluded due to inattentiveness.

Two looking tests (centrally presented faces, laterally presented faces) were conducted after twelve trials of the familiarization phase. During these tests, faces were presented alone on a white background, for twenty seconds each, with an approximate 20° distance between faces. The order of presentation of the looking tests and target locations were counterbalanced across participants. Five infants who did not look at the AOIs for at least two seconds were excluded.

### Analysis

To confirm that we had set up a mock IJA situation, we checked that infants looked first at the central face, then, shifted to the lateral face and then shifted back to the central face. At least five-hundred milliseconds duration of gaze fixation was required for each face to match the criteria for looking; however, after we excluded 10 participants due to inattentiveness, no trial was excluded on these criteria. Thus, all the trials were mock IJA situations.

We defined areas of interest (AOIs) of the same size in each situation. During the familiarization phase, two AOIs were defined: the centrally presented face (central face AOI) and the laterally presented face (lateral face AOI). There were two additional AOIs presented during each of the two looking-time tests (i.e., central face test and lateral face test): One was either the gazed-at face or the gazing face (JA condition), and the other was either the non-gazed-at face or the non-gazing face (no-JA condition). All the eye tracking data were recorded as percentages of looking time.

A fixation filter was required for the eye-tracking data; therefore, we used “Clearview” fixation filter and fixation was defined as follows: the eyes not shifting more than 50 pixels for at least 200 milliseconds. To determine the duration of fixation, this was applied to the raw eye-tracking data. The recorded sample’s average percentage was 72.6% (*SD* = 11.6, range: 52–94%). You can see all data in the **Supplementary Data Sheet S1**.

## Results

### Familiarization Phase

**FIGURE 2:**
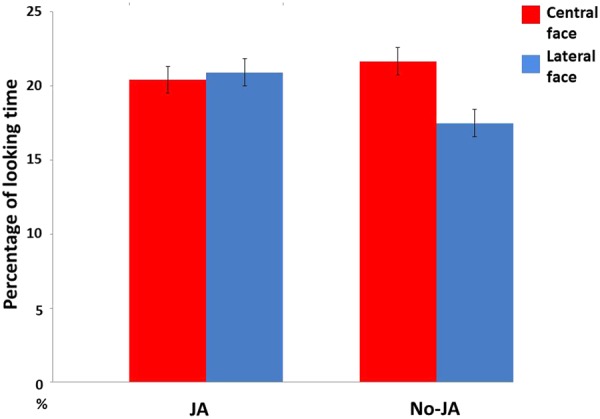
Mean percentages of looking time at four AOIs during the familiarization phase. The error bar indicates ± 1 standard error.

During the analysis of looking time during the familiarization phase, a 2 × 2 ANOVA with two levels of JA (JA, No-JA) and two levels of AOI (central face, lateral face) were conducted, to detect looking times after the central face shifted her gaze direction (5 s). The interaction effect of AOI and JA was not significant [*F*(1,18) = 3.45, *p* = 0.08, $\eta_{p}^{2}$ = 0.161, **Figure 2**]. It showed a significant trend, but no difference was observed after a *post hoc t*-test. The effect of AOI was not significant [*F*(1,18) = 0.56, *p* = 0.46, $\eta_{p}^{2}$ = 0.031]. The effect of JA was not significant [*F*(1,18) = 0.305, *p* = 0.58, $\eta_{p}^{2}$ = 0.017].

### Central Face Looking Test

**FIGURE 3:**
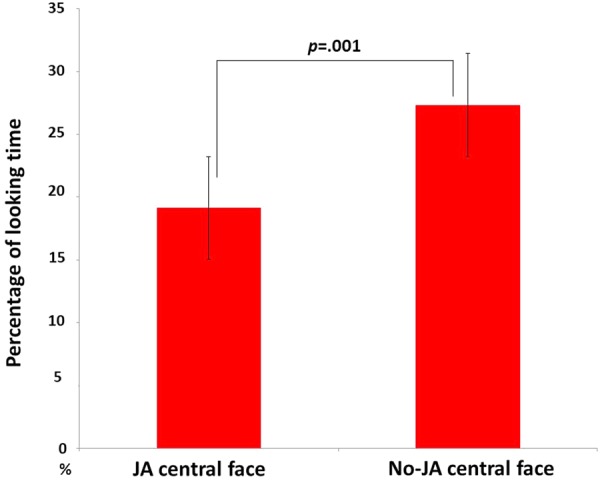
Mean percentages of looking time at two faces during the central face looking test. The error bar indicates ± 1 standard error.

The analysis of the looking test with the centrally presented faces revealed a significantly greater percentage of looking time at the no-JA condition (27.32%) than at the JA condition (19.14%; *t*(18) = -3.9, *p* = 0.001) (**Figure 3**).

### Lateral Face Looking Test

**FIGURE 4:**
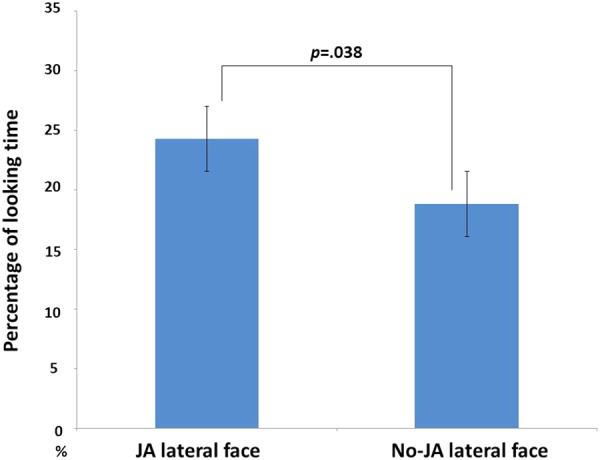
Mean percentages of looking time at two faces during the lateral face looking test. The error bar indicates ± 1 standard error.

The analysis of looking times at laterally presented faces revealed a significant difference between the JA and no-JA target faces [*t*(18) = 2.24, *p* = 0.038], with a greater percentage of looking time at the JA condition (24.29%) than the no-JA condition (18.82%) (**Figure 4**).

## Discussion

We investigated how another person’s shift of gaze affected facial preferences in infants, and we found that another person’s gaze shift influenced the preferences of infants. During the looking test phase, the JA targets – lateral faces that were gazed at by central faces – were looked at longer than the lateral faces that were gazed away from by the central faces. In addition, the JA gaze shifter, the central face that gazed at the lateral face, was looked at less than the no-JA gaze shifter.

During the familiarization phase, no significant effect of JA was observed. There was only a significant trend was observed in the interaction effect of AOI and JA. The whole design requires that infant see the different types of faces. The analyses show that infants looked at each face and shifted their gaze between them.

As predicted, the JA lateral face was looked at longer than the no-JA lateral face during the lateral face looking test. The social evaluation behavior of infants can explain this result. Human eye gaze can be a signal of intention, desire, social interest, and attraction (Mason et al., 2005; Gobel et al., 2015). Thus, faces looked at by the central faces may have been evaluated as attractive. However, previous studies using objects as gaze targets during looking tests indicated that target objects gazed at by others were looked at for shorter times than objects that others ignored (Reid and Striano, 2005; Okumura et al., 2013). This may suggest a preference for novelty objects. However, we found that infants preferred the target faces that the gaze shifter looked at. Liao et al. (2011) found that familiar face stimuli were preferred significantly more frequently than unfamiliar faces were. Therefore, our results may reflect differences in the target stimuli category, objects or faces.

During the central face looking test, the no-JA central face was looked at more than the JA central face was looked at, although we had predicted that infants would evaluate the JA central face to be more trustworthy than the no-JA central face (Bayliss and Tipper, 2006). This difference in looking time may reflect an expectancy violation. Ishikawa et al. (unpublished) revealed that infants looked longer at a JA gaze shifter during a mock IJA paradigm; however, the gaze targets employed in that were objects, not faces as in the present study. During the last scene of the present experiment’s initial trial phase, two faces were presented. If the infants had developed the expectation that humans interact, the no-JA gaze shifter would have violated this expectation and, therefore, would have been looked at longer. Beier and Spelke (2012) indicated that 10-month-old infants expect a person to look at their social partner, and this was supported by our finding. 10-month-old infants may already expect people to pay attention to others, and interact with others in social situations, including two or more humans. Further studies are required to examine the expectations of infants of social interactions.

The current study used a mock IJA paradigm, not simply observations of eye-gaze shifts. Although our paradigm only examined eye-gaze cuing effects on facial preferences, other effects of IJA would also be operating. It is difficult to distinguish whether only eye gaze affects facial preference or the whether the IJA situation has some effects. However, previous studies of JA suggested that an IJA situation has a stronger effect on preference than gaze cuing does (Grynszpan et al., 2017). Therefore, to maximize the effects of eye gaze, we adopted a mock IJA paradigm with infants. In future studies, a comparison between RJA and IJA effects upon infants may be required to clarify the effects of eye gaze.

This study had some limitations. First, the sample size was relatively small. Although we recruited about thirty babies, it was difficult to collect data from all of them. This experimental paradigm might be too long for 10-month-old infants; therefore, it should be improved to minimize the number of trials. Second, we did not use a gaze contingent paradigm to design our IJA situation. Previous studies have used IJA paradigms by using gaze-contingent programming (Grynszpan et al., 2017); however, we only used a mock IJA paradigm, to try and track infants following the gaze. However, adult studies with screen-based IJA situations have used gaze-contingent paradigms and instructed participants to look at one of two objects during the selection phase (Bayliss et al., 2013), whereas it is difficult to fix an infant’s gaze at one point only. In addition, a second-person framework may make it possible to examine the effects of IJA on infant cognition. IJA requires infants to use their gaze to direct or share others’ attention in the real social interactions, therefore second-person framework, which means that two real people interact via a monitor, may be helpful to examine the effects of IJA on infant cognition in experimental conditions. Schilbach et al. (2013) suggested that real-time interaction through a monitor permits investigations of social cognition such as JA (see also Pfeiffer et al., 2013). The present study is the very first to investigate how eye gaze directed toward another face affect face preferences in infants during a mock IJA situation. There are no infant JA studies using a second-person framework to compare IJA and RJA effects on infant cognition such as preferences or object processing; therefore, further studies should be conducted using gaze-contingent IJA paradigms or an interactive gaze paradigm between two real people and compare between effects of IJA and RJA.

We revealed that an IJA with human faces has different effects on the follow-up looking test than an IJA with objects does. Our study suggests that the components of triadic interaction play a role also in the effects of the gaze upon infant behavior. It has been suggested that gazing at humans can signal either group exclusion or acceptance (Wesselmann et al., 2012). Thus, all these components of a triadic interaction should be considered by JA studies.

## Data Availability

The data sets are available in the **Supplementary Data Sheet S1**.

## Author Contributions

MI conceived and designed the study, conducted the experiment, analyzed the data, and wrote the manuscript. SI participated in the data interpretation and helped to draft the manuscript. Both of the authors gave their final approval for publication.

## Conflict of Interest Statement

The authors declare that the research was conducted in the absence of any commercial or financial relationships that could be construed as a potential conflict of interest.

## Supplementary Material

DATA SHEET S1 All looking time data used for analysis.
